# HBcAb Positivity Is a Risk Factor for an Increased Detectability of HIV RNA after Switching to a Two-Drug Regimen Lamivudine-Based (2DR-3TC-Based) Treatment: Analysis of a Multicenter Italian Cohort

**DOI:** 10.3390/microorganisms9020396

**Published:** 2021-02-15

**Authors:** Vincenzo Malagnino, Elisabetta Teti, Mirko Compagno, Luigi Coppola, Romina Salpini, Valentina Svicher, Monica Basso, Giuliana Battagin, Sandro Panese, Maria Cristina Rossi, Renzo Scaggiante, Daniela Zago, Marco Iannetta, Saverio Giuseppe Parisi, Massimo Andreoni, Loredana Sarmati

**Affiliations:** 1 Clinica di Malattie Infettive, Policlinico Tor Vergata di Roma, 00133 Rome, Italy; elisabetta.teti@gmail.com (E.T.); mirkocompagno2@gmail.com (M.C.); luigi.coppolamed@gmail.com (L.C.); marco.iannetta@uniroma2.it (M.I.); andreoni@uniroma2.it (M.A.); srmldn00@uniroma2.it (L.S.); 2 Dipartimento di Medicina dei Sistemi, Facoltà di Medicina, Università degli studi di Roma Tor Vergata, 00133 Rome, Italy; 3 Dipartimento di Medicina Sperimentale, Università degli Studi di Roma Tor Vergata, 00133 Rome, Italy; rsalpini@yahoo.it (R.S.); valentina.svicher@uniroma2.it (V.S.); 4 Dipartimento di Medicina Molecolare, Università degli Studi di Padova, 35128 Padova, Italy; monica.basso@unipd.it (M.B.); daniela.zago.3@gmail.com (D.Z.); saverio.parisi@unipd.it (S.G.P.); 5 UOC Malattie Infettive, Ospedale di Vicenza, 36100 Vicenza, Italy; giuliana.battagin@aulss8.veneto.it; 6 UOC Malattie Infettive Ospedale di Venezia, 30122 Venezia, Italy; sandro.panese@aulss3.ve.it; 7 UOC Malattie Infettive Ospedale di Treviso, 31100 Treviso, Italy; mariacristina.rossi@aulss2.veneto.it; 8 UOC Malattei Infettive, Ospedale di Belluno, 32100 Belluno, Italy; renzo.scaggiante@aulss1.veneto.it

**Keywords:** anti-HBc, HIV/HBV coinfection, 2DR, 3TC/DTG, dual therapy

## Abstract

The aim of this study was to evaluate whether the presence of anti-hepatitis B (HBV) c antibodies (HBcAb positivity) could influence the control of Human Immunodeficiency Virus (HIV) viremia in patients living with HIV (PLWH) who switch a to two-drug antiretroviral therapy (2DR) containing lamivudine (3TC) (2DR-3TC). A retrospective observational multicenter study was conducted on 166 PLWH switching to the 2DR-3TC-based regimen: 58 HBcAb-positive and 108 HBcAb-negative patients. The HBcAb-positive PLWH group demonstrated a significantly higher percentage of subjects with very low-level viremia at all time points after switching (6th month: <31% vs. 17.6%, *p* = 0.047; 12th month 34% vs. 27.5%, *p* = 0.001; 24th month 37% vs. 34.2, *p* = 0.003 of the HBcAb-positive and HBcAb-negative groups, respectively) and a higher percentage of subjects with detectable HIV RNA greater than 20 copies/mL 12 and 24 months after switching (12 months 32% vs. 11%, *p* = 0.001; 24 months 37% vs. 13.9%, *p* = 0.003 of the HBcAb-positive and HBcAb-negative groups, respectively). Logistic regression analysis showed that an increase in age of ten years (OR 2.48 (95% CI 1.58–3.89), *p* < 0.0001) and the presence of HBcAb positivity (OR 2.7 (5% CI 1.05–6.9), *p* = 0.038) increased the risk of detectability of HIV RNA by nearly three-fold after switching to 2DR-3TC.

## 1. Introduction

Occult B infection (OBI) is characterized by the persistence of hepatitis B virus (HBV) DNA in the liver, regardless of its presence in serum. Patients with OBI may or may not have serological signs of a previous HBV infection; however, the presence of antibodies against an HBV c antigen (HBcAb) is usually considered a surrogate marker of an OBI condition [1]. Immunosuppression is often related to a deficiency in the HBV immune response resulting in the development of chronic or occult HBV infection, and cancers, immunosuppressive treatments, and human immunodeficiency virus (HIV) infection are known to predispose patients to OBI [2]. OBI is quite common in patients living with HIV (PLWH), particularly in countries with a high HBV prevalence [3,4], and low CD4+ counts or AIDS-related diseases are more common in PLWH with OBI treated or not with antiretroviral therapy (ART) [5,6].

The current triple ART includes HBV-active drugs in its composition; the choice of a third drug with potent anti-HBV activity, such as tenofovir (TDF) or tenofovir alafenamide (TAF), is strongly recommended in the treatment of HIV/HBV-coinfected patients [7]. In PLWH subjects with OBI, it has been shown that the initiation of ART containing lamivudine (3TC) or TDF achieves the durable suppression of HBV-DNA [8], and occasional cases of HBV reactivation in PLWH subjects with OBI have been reported after discontinuation of the anti-HBV drug [9,10]. Moreover, treatment of HIV/HBV-coinfected patients with 3TC in the ART composition, once widely carried out in resource-limited settings, is currently not recommended due to the rapid loss of drug efficacy owing to the emergence of resistant strains in most subjects [11,12].

In our recent paper, we demonstrated that HBcAb positivity was associated with a delay in achieving HIV undetectability and the onset of viral rebound in HBcAb-positive PLWH after triple ART initiation [13], thus suggesting the possible contribution of transient reactivation of HBV to poor HIV control. The presence of detectable HBV-DNA was demonstrated in 15% of ART-treated HIV/HBcAb-positive patients from an Italian cohort [14], and the presence of “cryptic” HBV viremia (below 20 IU/mL of commercial tests) was demonstrated in 29% of HBcAb-positive PLWH receiving ART [15].

In recent years, interest has grown in two-drug ART regimens designed to improve adherence and compliance by reducing ART toxicity but maintaining its efficacy. Many of these therapies are considered to be nucleoside reverse transcriptase inhibitor (NRTI)-sparing treatments, which essentially means that they eliminate HBV active molecules or, in some cases, use 3TC as a second drug, which is no longer recommended for HBV treatment. In HBsAg-positive PLWH, the use of NRTI-sparing treatments or a two-drug ART regimen with 3TC is not recommended; however, to date, no restrictions have been instituted regarding switching HBcAb-positive PLWH to dual-therapy.

We retrospectively evaluated HIV viremia values two years before and two years after the switch to two-drug antiretroviral therapy (2DR) containing 3TC (2DR-3TC) in a cohort of HBcAb-positive and HBcAb-negative PLWH to assess whether the elimination of HBV active drugs or their replacement with 3TC worsened the control of HIV viremia in HBcAb-positive PLWH.

## 2. Materials and Methods

### 2.1. Study Design

A retrospective observational study was conducted on 187 PLWH switched to the 2DR-3TC-based regimen and followed up thereafter. Patients were enrolled at different clinical sites: The Infectious Diseases Clinic of the Tor Vergata Polyclinic in Rome, the Department of Molecular Medicine of the University of Padua, the Units of Infectious Diseases of Vicenza, and the Venezia and Treviso hospitals. For this study, a database was built which included all patients’ data at HIV infection diagnosis (baseline), at the start of ART treatment, and at follow-up visits. In particular, the following data were collected for all patients: demographic information; HBV serology at baseline; CD4+ cell count at baseline and at the time of the 2DR-3TC switch; calendar year of the HIV infection diagnosis; ART composition before the 2DR-3TC switch; and HIV-RNA viral load at 6, 12, 18, and 24 months before the 2DR-3TC switch, at the time of the switch, and at 6, 12, 18, and 24 months after.

### 2.2. Inclusion and Exclusion Criteria

**Figure 1:**
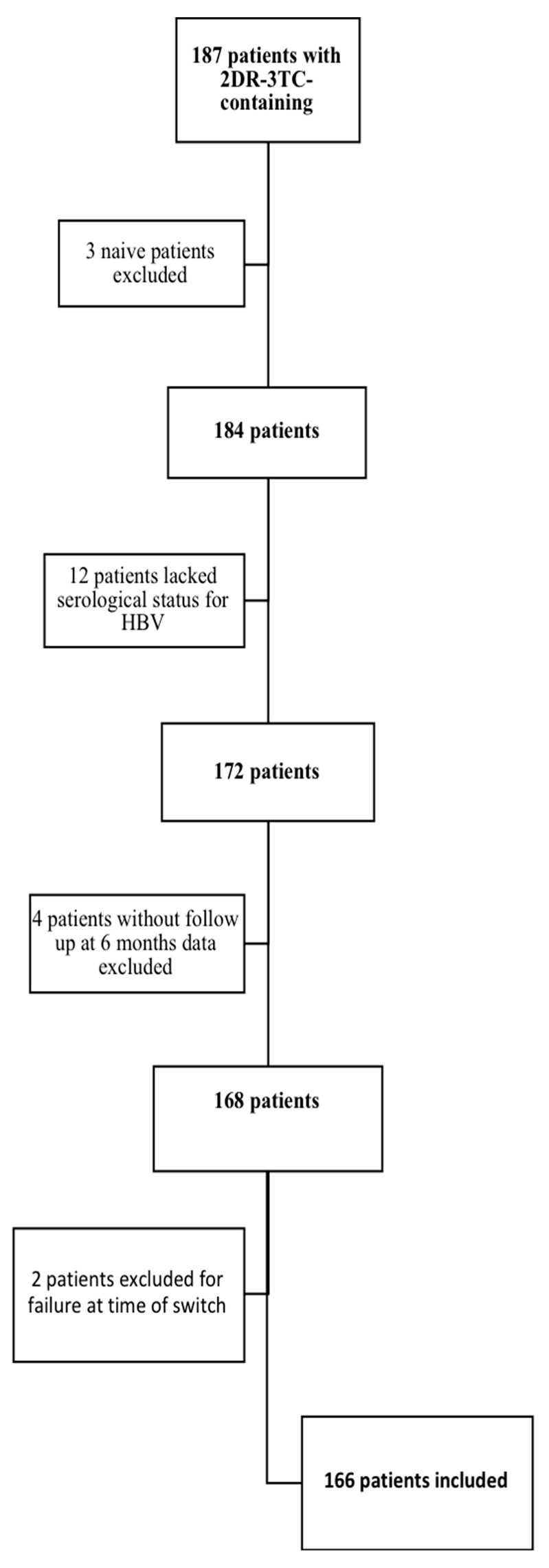
Patient inclusion algorithm.

As shown in Figure 1, of the 187 patients followed in the multicenter cohort, three patients were excluded because they started the 2DR-3TC regimen as naïve, twelve patients were excluded due to a lack of HBV serological data, two patients were excluded because they were in virologic failure at the time of the switch, and four patients were excluded because their virological data were missing at 6 and 12 months after the switch. Ultimately, 166 patients were studied.

### 2.3. Laboratory Testing for the Diagnosis of HBV and HIV Infections

HBV serological markers were measured using immune-enzymatic assays (Roche/Cobas Diagnostics, Rotkreuz, Switzerland).

Plasma HIV-RNA levels were measured using a commercial test with 20 cp/mL HIV RNA as the lower limit of quantification (COBAS AmpliPrep/COBAS TaqMan HIV-1 Test, v. 2.0). The test allows for the detection of the presence of HIV RNA without quantifying it under 20 copies/mL, which is defined as HIV-RNA detected or target detected [16].

### 2.4. HIV Viral Load Definitions

All patients enrolled in the study had an undetectable HIV viral load (<20 copies/mL, target not detected) at the time of the switch to 2DR-3TC treatment. Levels of HIV RNA detectable below the limits of commercial assays have been found to be associated with an increased risk of virological failure, virological rebound, and the development of resistance [17,18,19]. To evaluate the HIV viremia course after the 2DR switch in the study cohort, all HIV viral load measurements carried out at 6, 12, and 24 months before and after the transition to the 2DR-3TC regimen were considered. Based on the HIV-RNA assay results, the following definitions were adopted: (1) viral load undetectable (if viremia was less than 20 copies/mL, target not detected); (2) very low-level viremia (if viremia was below 20 copies/mL, target detected); and (3) viral load detectable (if viremia was above 20 copies/mL). Viremia values greater than 20 copies/mL or less than 20 copies/mL with targets detected were considered signs of active HIV replication [16].

### 2.5. Endpoints

The endpoint of the study was to evaluate differences in the maintenance of HIV viremia suppression between the two PLWH populations of HBcAb-positive and HBcAb-negative patients 6, 12, and 24 months after the 2DR-3TC-based switch.

### 2.6. Statistical Methods

The data were collected, and the dataset was assembled using Excel 2019 v. 16.3. All statistical analyses were conducted using STATA 14.2 (College Station, TX, USA), and graphs and confirmation analyses were performed using GraphPad Prism.v. 8.2.1.

The study population is described using proportions and percentages for categorical values and median measurements and interquartile ranges (IQRs) for continuous values. The comparison between HBcAb-positive PLWH and HBcAb-negative PLWH was performed with the Kruskal–Wallis test for continuous variables and with the chi-squared test or Fisher’s exact test, when appropriate, for categorical variables. Univariable odds ratios (ORs) associated with a lack of HIV-RNA undetectability at T24 and their 95% confidence intervals (CIs) were calculated using logistic regression. A multivariable model was constructed in which risk factors with *p* < 0.1 in univariable analysis were included in a full model and were then excluded in a backwards-stepwise fashion if *p* < 0.05 using the likelihood ratio test.

### 2.7. Ethic Statements

The study protocol and related informed consent were submitted and approved by the Independent Ethics Committee at Policlinico Tor Vergata (Protocol Number 216/16, version 1.0); the study was structured with respect to privacy. Personal information was treated in a confidential manner, and clinical data were anonymized in accordance with the Helsinki Declaration (October 2013 version). All included patients signed an informed consent for inclusion in the observation.

## 3. Results

### 3.1. Description of the Study Population

The full description of the study population is shown in Table 1. One hundred sixty-six patients who switched to the 2DR-3TC regimen were studied; 38 were female (22.9%), the median age was 52.5 years (IQR 42-59), and the median duration of HIV infection was approximately ten years (calendar year of HIV infection 2009 (IQR 2003–2013)). Regarding the lymphocyte subpopulations, the CD4+ cell nadir median value was 293/mm^3^ (IQR 172–414), while the median CD4+ cell count at the time of the switch was 695/mm^3^ (IQR 547–880). Approximately one-third of the patients (58/166, 34.9%) tested positive for HBcAb. Triple ART treatment before the switch to 2DR-3TC showed a balance in the choice of drug classes used: 65 patients (39.2%) were on two NRTIs plus a protease inhibitor (PI), 38 (22.9%) were on two NRTIs plus a non-nucleoside reverse transcriptase inhibitor (NNRTI), 56 (33.7%) were on two NRTIs plus an integrase inhibitor (INI), and 7 (4.2%) were on other non-conventional therapeutic regimens (four-drug therapy or triple ART with PI + INI + 3TC). Most patients (130, 78.3%) reached viral load undetectability 24 months after the start of triple ART, with three adjunctive subjects at 12 months (133, 80.1%). At the time of the switch to 2DR treatment, 36 (31.7%) subjects showed a very low level of viremia (<20 copies/mL with a target detected).

#### HIV Viral Load Values at the Different Time Points After Switching to the 2DR-3TC Regimen

The 2DR-3TC simplification schemes were distributed as follows: 59 patients (35.5%) moved to 3TC plus PI (atazanavir (ATV) or darunavir (DRV) regardless of whether boosted with ritonavir (RTV) or cobicistat (COBI)), and 107 (64.5%) moved to 3TC plus dolutegravir (DTG). All patients maintained virological suppression at 6 and 12 months after the switch, although with different proportions of subjects with very low-level viremia: at the 6th month, 77.7% had <20 copies and no detectable target, while at the 12th month (data available for only 144 patients), only 50.4% had <20 copies and no detectable target, almost 30% had a very low level of viremia, and 18.7% showed a detectable viremia > 20 copies/mL. At month 24 (data available only for 125 patients), the proportion of patients with a very low level of viremia grew to 35.2%, while 22.4% of subjects were viremic with HIV-RNA load values above 20 copies/mL.

### 3.2. Comparison of HIV Viral Load Values between HBcAb-Positive and HBcAb-Negative Patients

#### 3.2.1. HIV Viral Load Values at the Different Time Assessments in the Two Groups before the 2DR-3TC Switch

A comparison between HBcAb-negative and HBcAb-positive patients in the study cohort is reported in Table 2. HBcAb-positive patients tended to be older (55 years (IQR 51–61), *p* = 0.0007) and had a longer history of HIV infection (2002 and 2008, respectively, in HBcAb-positive and HBcAb-negative patients, *p* = 0.0001) and were likely more frequently on PI-containing triple ART regimens (53.4% vs. 31.5%, respectively, of HBcAb-positive and HBcAb-negative subjects, *p* = 0.023). Conversely, HBcAb-negative patients were more frequently treated with regimens containing INIs (40.7% vs. 20.7%, respectively, of HBcAb-negative and HBcAb-positive subjects). No statistically significant difference in switch reasons was found in the study population (*p* = 0.44), furthermore, in both subpopulations the main reason was the medical choice of pre-emptive switch (43 HBcAb-positive patients (74.1%) and 70 HBcAb-negative patients (64.8%)).

The HBcAb-positive group showed a significantly lower CD4+ cell nadir value (251 cells/mm^3^ (IQR 145–341) versus 316 cells/mm^3^ (IQR 201–446), respectively, in the HBcAb-positive and HBcAb-negative groups, *p* = 0.047); however, there was no difference in CD4+ cell count between the two groups of patients at the time of switching to 2DR-3TC (*p* = 0.58). The proportions of subjects with a history of therapeutic failure were similar, and no differences in the proportions of patients with different viremia determinations (below 20 copies/mL, target not detected; below 20 copies, target detected; and above 20 copies/mL) were observed in the two groups 24 and 12 months before the switch and at the time of simplification to 2DR-3TC (*p* = 0.53; *p* = 0.31; and *p* = 0.87, respectively).

#### 3.2.2. HIV Viral Load Values at the Different Time Assessments in the Two Groups after the 2DR-3TC Switch

No difference in the 2DR-3TC composition was observed in the two groups of patients, and higher proportions of patients in both groups resulted in a 2DR-3TC regimen containing DTG (58.6% and 67.6%, respectively, of HBcAb-positive and HBcAb-negative patients). Less control of HIV replication was demonstrable in the HBcAb-positive group at all time point assessments after simplification. In the HBcAb-positive group, a higher proportion of subjects with a very low level of viremia was demonstrated six months after the switch (<20 copies/mL target detected 31% vs. 17.6%, respectively, in the HBcAb-positive and HBcAb-negative groups, *p* = 0.047), and a progressive reduction of patients with undetectable viremia was present at all time points (<20 copies/mL, target not detected at the 6th month, 69% vs. 82.4%, *p* = 0.047; at the 12th month, 34% vs. 61.5%, *p* = 0.001; and at the 24th month, 26% vs. 51.9%, *p* = 0.003, respectively, of the HBcAb-positive and HBcAb-negative groups). Moreover, progressive increases in patients with detectable viremia above 20 copies/mL were shown after 12 months (32% vs. 11%, respectively, of the HBcAb-positive and HBcAb-negative groups, *p* = 0.001) (data available for 144 patients) and 24 months (37% vs. 13.9%, respectively, of the HBcAb-positive and HBcAb-negative groups, *p* = 0.003) (data available for 125 patients).

To assess whether the use of INIs in the 2DR-3TC compositions could have influenced the results of the comparison, the analysis was performed only for the subgroups of patients on 2DR-3TC plus DTG. Thirty-four HBcAb-positive and 78 HBcAb-negative PLWH treated with 2DR-3TC plus DTG were evaluated. Significantly lower proportions of subjects with HIV-RNA load <20 copies/mL, target not detected, were demonstrated in the HBcAb-positive group 12 months (32.3% versus 63.8% in the HBcAb-positive and HBcAb-negative groups, respectively, *p* = 0.02) and 24 months (20.8% versus 54.2% in the HBcAb-positive and HBcAb-negative groups, respectively, *p* = 0.006) after the switch. In addition, a higher proportion of subjects in the HBcAb-positive group showed very low-level viremia (target detected below 20 copies/mL, 35.4% vs. 24.1% after 12 months and 37.5% vs. 33.3% after 24 months, respectively, in HBcAb-positive and HBcAb-negative subjects) and a detectable viremia above 20 copies (32.3% vs. 12.1% after 12 months and 41.7% vs. 12.5% after 24 months, respectively, in the HBcAb-positive and HBcAb-negative groups) (*p* = 006). No difference in viremia values were detected between the two groups of patients at six months from the switch (data showed in Table S1).

#### 3.2.3. HIV Viral Load Values in HBcAb -Positive and HBcAb -Negative Patients with HIV RNA Permanently Undetectable to All Assessments before 2DR-3TC Switching

**Figure 2:**
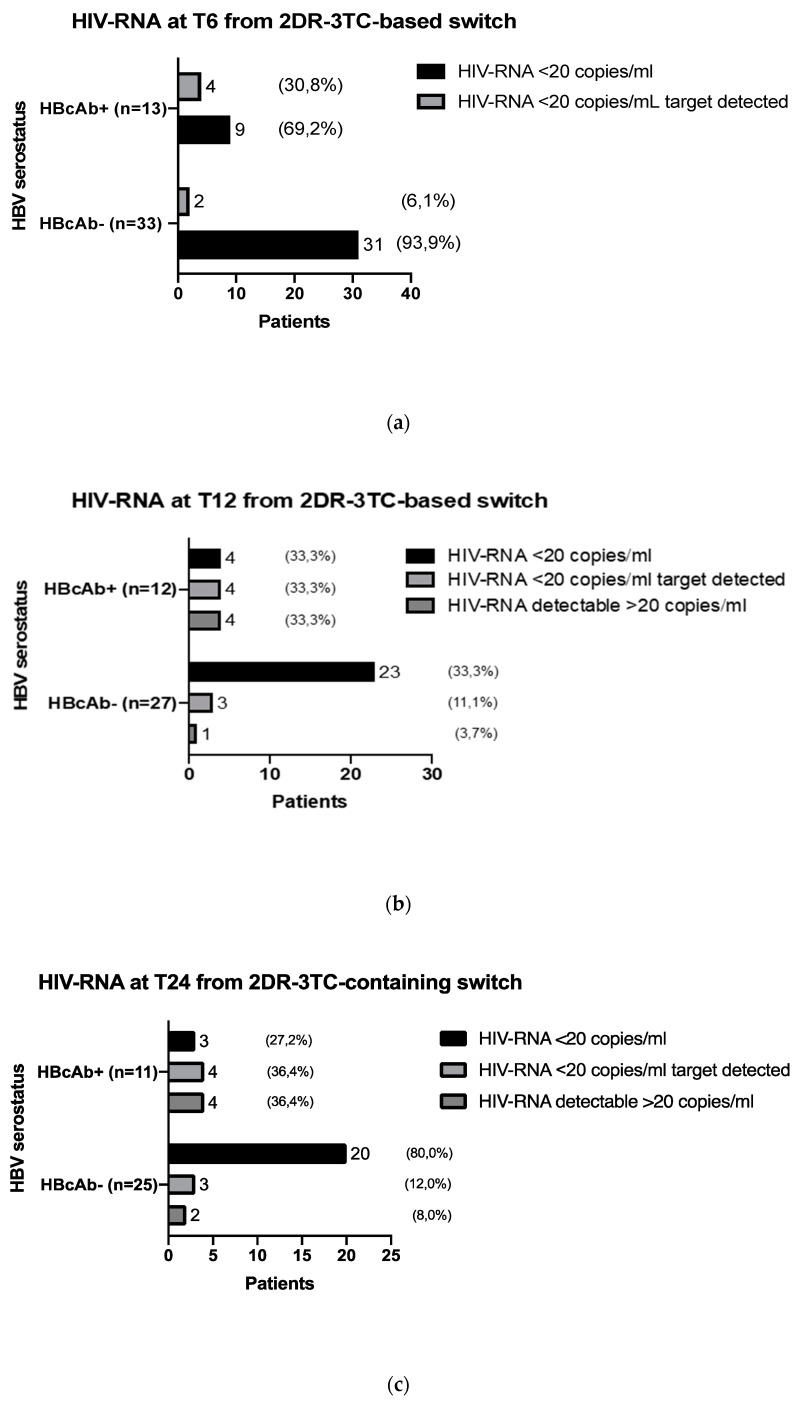
Comparison of HIV-RNA undetectability after the 2DR-3TC switch in the HBcAb-positive and HBcAb-negative groups, after 6 months (**a**) T6, *n* = 46, 12 months, (**b**) T12, *n* = 39, and 24 months (**c**) T24, *n* = 36, and overall comparison of patients subgroup characteristics.

To exclude possible confounding factors, we evaluated HIV-RNA values at 6, 12, and 24 months from the switch to 2DR-3TC in patients with viremia stably not detectable in the 24 months preceding the therapeutic change and without a previous history of virological failure. The numbers of evaluable study cohort patients were 46 at the 6th month, 39 at the 12th month, and 36 at the 24th month; the results of the analysis are shown in Figure 2 and Table 3. Compared with the HBcAb-negative patients, a significantly higher number of subjects in the HBcAb-positive group had low-level viremia at all time points, and significantly more HBcAb-positive patients had detectable viremia at months 12 and 24.

#### 3.2.4. Risk Factors for Signs of HIV-RNA Detectability 24 Months after the 2DR-3TC-Based Switch

A logistic regression analysis (Table 4) was performed with the production of a multivariate model including age (considered as a ten-year increase), calendar year of HIV infection, nadir CD4+ cell count (considered as an increase of 250 cells/mm^3^), switch to 2DR-3TC containing DTG, and HBcAb positivity. The analysis showed that 24 months after simplification to a 2DR-3TC regimen, a ten-year increase in age (OR 2.48 (95% CI 1.58–3.89), *p* < 0.0001) and the presence of HBcAb positivity (OR 2.7 (95% CI 1.05–6.9), *p* = 0.038) increased the risk of HIV-RNA detectability more than two-fold (very low-level viremia and detectable viremia above 20 copies/mL).

## 4. Discussion

In our cohort of virologically suppressed PLWH who switched to a 2DR-3TC regimen, HBcAb positivity was significantly associated with the worst HIV replicative control at all time points of follow-up and correlated with a nearly three-fold increase in the risk of HIV-RNA detectability at 24 months after the 2DR-3TC switch (OR 2.7 (95% CI 1.05–6.9), *p* = 0.038).

Achieving HIV suppression below the detection limits of laboratory tests remains the main goal of ART, and its maintenance throughout the duration of therapy is the best guarantee of its effectiveness. However, the observation of occasional or persistent viremia above the test threshold in patients on ART is common and has often been associated with virologic failure [10,18,20,21]. Additionally, in our HIV-HBcAb-negative study population, the presence of very low-level viremia occurred in 18% of subjects at 6 months, 27% at 12 months, and 34% at 24 months of ART, while detectable viremia above 20 copies/mL was found in 11% of patients at 12 months and 14% at 24 months. Few data are available in the literature on the correlation between viremia above or below 20 copies/mL and the onset of virological failure. Laprise et al. [22] described that persistent HIV-RNA detection below 50 copies/mL at 1, 2, and 5 years after ART initiation is associated with 5-, 10-, and 22-fold cumulative risks of virologic failure, respectively. In addition, Maggiolo and colleagues [18] demonstrated that HIV-RNA detection above 3 copies/mL is predictive of virological failure.

In a previous study [13], we described how HBcAb positivity was associated with a delay in achieving virological success and more frequent virological failure in HIV/HBV-coinfected patients starting ART, and we argued that possible occasional HBV replication, common in OBI patients, could contribute to the poor control of HIV replication, as demonstrated in other viral coinfections (i.e., cytomegalovirus and hepatitis C virus). The occasional presence of detectable HBV-DNA in the blood of PLWH with OBI is widely documented [4,14,15], and in vitro studies have shown the ability of HBV to facilitate HIV replication. Twu JS and Gomez-Gonzalo M and colleagues [23,24] demonstrated that the HBV X gene induces HIV-1 replication and HIV-1 long terminal repeat (LTR) transcription by synergizing with the Tat protein. Moreover, a higher degree of immune activation, contributing to HIV replication, was demonstrated in HIV/HBV-coinfected patients, and higher plasma soluble CD14 levels and increased percentages of CD4+/HLA-DR+/CD38+ and CD8+/HLA-DR+/CD38+ T lymphocytes have been demonstrated in HIV/HBV-coinfected subjects compared with those infected with either HBV or HIV alone [21,25].

Studies on HBcAb-positive PLWH treated with a 2DR regimen are lacking, and published papers on HIV/HBV ART simplified subjects are mainly focused on HBV replication control. Abdullahi et al. [26] described the evolution of HBV markers among patients switching to an NRTI-sparing regimen (mainly PI monotherapy), and 10% of HBcAb-positive PLWH (109 cases/1000 person-years) showed HBV reactivation.

In this regard, it is emphasized that all patients whose HIV viremia was poorly controlled during the 2DR-3TC-based switch did not undergo therapeutic failure, the appearance of resistance mutations, or the onset of AIDS events. Furthermore, of the patients included in the study, 15 returned to triple therapy more than 24 months after the switch unrelated to the aforementioned reasons.

Regarding the choice of 3TC in simplified treatment schemes, its use has been proven to be ineffective in the control of HBV replication even in HIV HBcAb-positive patients treated with triple ART. A recent South African prospective study investigating the impact of 3TC-containing ART regimens on the trend of HBV viremia during 24 months of follow-up demonstrated that 25% of patients with proven OBI had detectable HBV DNA at 24 months after the introduction of 3TC [12]. In addition, the possibility of HBV-immune escape mutant emergence is a well-known phenomenon in patients treated with 3TC [2].

Before drawing conclusions, a number of study limitations need to be stated. First, the retrospective nature of this study may introduce selection and information biases, such as lack in follow-up data or anamnestic data, in particular HCV serostatus and risk factors for HIV transmission. Second, the reasons for switching (pre-emptive switch, drug interaction, drug toxicity, or cost) are not homogeneous for all patients, and different simplification schemes were taken into consideration (PI- or INI-based). Third, we did not evaluate ART adherence, and the follow-up of patients in the present study might have been too short to observe treatment failures.

In conclusion, to the best of our knowledge, this is one of the first studies investigating the role of HBcAb positivity in the control of HIV RNA in PLWH switched to 2DR-3TC. Based on the presented results, the condition of potential OBI should be carefully considered when selecting PLWH candidates for 2DR switching, as they are potentially at risk of possible future therapeutic failure. In addition, an increase in serological screening of HBV infection and periodic HBV-DNA monitoring in HBcAb-positive PLWH should be encouraged to better monitor occasional HBV viremia appearance and to prevent frank HBV reactivation.

## Figures and Tables

**Table 1:** Characteristics of the study population.

Total Population		*n* = 166
Sex ratio M:F (%F *)		128:38 (22.9%)
Age, years **		52.5 (42–59)
Calendar year of HIV diagnosis **		2009 (2003–2013)
Nadir CD4+, cell/mmc **		293 (172–414)
HBcAb-positive *		58 (34.9%)
CD4+ cell/mmc at 2DR switch **		695 (547–880)
Triple ART drug classes composition *:		
	Two NRTIs + PI	65 (39.2%)
	Two NRTIs +NNRTI	38 (22.9%)
	Two NRTIs + INI	56 (33.7%)
	Other	7 (4.2%)
2DR composition *:		
	3TC + PI	59 (35.5%)
	3TC + DTG	107 (64.5%)
HIV-RNA copies/mL 24 months before 2DR switch *:		
	pts <20 copies/ml	130 (78.3%)
	pts <20 copies/mL target detected	36 (21.7%)
HIV-RNA copies/mL 12 months before 2DR switch *:		
	pts <20 copies/ml	133 (80.1%)
	pts <20 copies/mL target detected	33 (19.8%)
HIV RNA at 2DR switch *:		
	pts <20 copies/ml	126 (75.9%)
	pts <20 copies/mL target detected	36 (31.7%)
	Not available	4 (2.4%)
HIV RNA 6 months post-2DR switch *		
	pts <20 copies/ml	129 (77.7%)
	pts <20 copies/mL target detected	37 (22.3%)
HIV RNA 12 months post-2DR switch *^††^:		
	pts <20 copies/ml	74 (50.4%)
	pts <20 copies/mL target detected	43 (29.9%)
	pts detectable >20 copies/ml	27 (18.7%)
HIV RNA 24 months post-2DR switch *^‡^:		
	pts <20 copies/ml	53 (42.4%)
	pts <20 copies/mL target detected	44 (35.2%)
	pts detectable >20 copies/ml	28 (22.4%)

* Number (%), ** Median (IQR). 3TC: lamivudine; NRTIs: nucleoside reverse transcriptase inhibitors; NNRTI: nonnucleoside reverse transcriptase inhibitor; PI: protease inhibitor; DTG: dolutegravir; 2DR: two-drug antiretroviral therapy; pts: patients; HBcAb-positive: patients with antibodies against hepatitis B c antigen. ^††^ Data available for 144 pts; ^‡^ Data available for 125 pts.

**Table 2:** Comparison of HIV viral load values at the different time points between the HBcAb-positive patients living with HIV (PLWH) and HBcAb-negative PLWH groups.

Patients Characteristics		HBcAb-Positive (*n* = 58)	HBcAb-Negative (*n* = 108)	*p*-Value
Sex ratio M:F (%F *)		46:12 (20.7%)	82:26 (24.1%)	0.62
Age, years		55 (51–61)	49 (37–57)	**0.0007**
Calendar year of HIV diagnosis **		2002 (1999–2010)	2008 (2006–2014)	**0.0001**
Nadir CD4+ cell/mmc **		251 (145–341)	316 (201–446)	**0.047**
History of therapeutic failure *		12 (20.7%)	12 (11.1%)	0.26
Pre-2DR switch triple ART composition *				**0.023**
	Two NRTIs + PI	31 (53.4%)	34 (31.5%)	
	Two NRTIs + NNRTI	12 (20.7%)	26 (24.1%)	
	Two NRTIs + INI	12 (20.7%)	44 (40.7%)	
	Other	3 (5.2%)	4 (3.7%)	
Reason for switch				0.44
	Therapeutic failure pre-switch	1 (1.7%)	2 (1.85)	
	Toxicity	4 (6.8%)	10 (9.2%)	
	Drug interaction	7 (12.1%)	17 (15.7%)	
	Pill burden	3 (5.2%)	9 (8.3%)	
	Pre-emptive switch	43 (74.1%)	70 (64.8%)	
2DR composition *				0.51
	3TC + PI	24 (41.4%)	35 (32.4%)	
	3TC + DTG	34 (58.6%)	73 (67.6%)	
CD4+ cell/mmc at 2DR switch **		660 (552–845)	703 (543–894)	0.58
HIV RNA 24 months pre-2DR switch *				0.53
	pts <20 copies/mL	47 (81%)	83 (76.8%)	
	pts <20 copies/mL target detected	11 (19%)	25 (23.2%)	
HIV RNA 12 months pre-2DR switch *				0.31
	pts <20 copies/mL	44 (75.9%)	89 (82.4%)	
	pts <20 copies/mL target detected	14 (24.1%)	19 (17.6%)	
HIV RNA at 2DR switch *				0.87
	pts <20 copies/mL	43 (74.1%)	83 (76.8%)	
	pts <20 copies/mL target detected	13 (22.4%)	23 (21.3%)	
	Not available	2 (1.8%)	2 (3.4%)	
HIV RNA 6 months post-2DR switch*				**0.047**
	pts <20 copies/mL	40 (69%)	89 (82.4%)	
	pts <20 copies/mL target detected	18 (31%)	19 (17.6%)	
HIV RNA 12 months post-2DR switch *^††^				**0.001**
	pts <20 copies/mL	18 (34%)	56 (61.5%)	
	pts <20 copies/mL target detected	18 (34%)	25 (27.5%)	
	pts detectable >20 copies/mL	17 (32%)	10 (11%)	
CD4+ cell/mmc at 24 months post 2DR switch *^‡^		698 (592–932)	771 (669–947)	0.64
HIV RNA 24 months post-2DR switch *^‡^				**0.003**
	pts <20 copies/mL	12 (26%)	41 (51.9%)	
	pts <20 copies/mL target detected	17 (37%)	27 (34.2%)	
	pts detectable >20 copies/mL	17 (37%)	11 (13.9%)	

* Number (%); ** Median (IQR); 3TC: lamivudine; NRTIs: nucleoside reverse transcriptase inhibitors; NNRTI: nonnucleoside reverse transcriptase inhibitors; PI: protease inhibitors; DTG: dolutegravir; 2DR: two-drug antiretroviral therapy; pts: patients; HBcAb-positive: patients with antibodies against hepatitis B c antigen. ^††^ Data available for 144 pts; ^‡^ Data available for 125 pts; bold numbers if *p*-value < 0.05.

**Table 3:** Comparison of HIV-RNA values after the 2DR-3TC switch in the HBcAb-positive and HBcAb-negative groups, after 6 months (T6, *n* = 46), 12 months (T12, *n* = 39), and 24 months (T24, *n* = 36), and overall comparison of patients subgroup characteristics.

Patients Characteristics		HBcAb-Positive (*n* = 13)	HBcAb-Negative (*n* = 33)	*p*-Value
Sex ratio M:F (%F *)		12:1 (7.7%)	22:11 (33.3%)	**0.07**
Age, years		55 (52–59)	46.5 (33.5–55.5)	**0.008**
Calendar year of HIV diagnosis **		1999 (1996–2006)	2009 (2007–2014)	**0.0005**
Nadir CD4+ cell/mmc **		208 (155–290)	355 (224–466)	**0.01**
Pre-2DR switch triple ART composition *				0.37
	Two NRTIs + PI	7 (53.8%)	12 (36.4%)	-
	Two NRTIs + NNRTI	4 (30.7%)	7 (21.2%)	-
	Two NRTIs + INI	2 (15.4%)	13 (39.4%)	-
	Other	0 (0)	1 (3%)	-
2DR Composition *				0.7
	3TC + PI	6 (46.2%)	11 (33.3%)	-
	3TC + DTG	7 (53.8%)	22 (66.7%)	-
CD4+ cell/mmc at 2DR switch **		671 (563–763)	726 (514–885)	**0.01**
HIV RNA at 2DR switch *				0.26
	pts <20 copies/mL	11 (84.6%)	31 (93.9%)	-
	pts <20 copies/mL target detected	1 (7.7%)	2 (6.1%)	-
	Not available	1 (7.7%)	0 (0)	-
HIV RNA 6 months post-2DR switch *				**0.025**
	pts <20 copies/mL	4 (30.7%)	2 (6.1%)	-
	pts <20 copies/mL target detected	9 (69.3%)	31 (93.9%)	-
HIV RNA 12 months post-2DR switch *^††^				**0.004**
	pts <20 copies/ml	4 (33.3%)	23 (85.2%)	-
	pts <20 copies/mL target detected	4 (33.3%)	3 (11.1%)	-
	pts detectable >20 copies/ml	4 (33.3%)	1 (3.7%)	-
HIV RNA 24 months post-2DR switch *^‡^				**0.009**
	pts <20 copies/ml	3 (27.2%)	20 (80%)	-
	pts <20 copies/mL target detected	4 (36.4%)	3 (12%)	-
	pts detectable >20 copies/mL	4 (36.4%)	2 (8%)	-

* Number (%); ** Median (IQR); 3TC: lamivudine; NRTIs: nucleoside reverse transcriptase inhibitors; NNRTI: nonnucleoside reverse transcriptase inhibitors; PI: protease inhibitors; DTG: dolutegravir; 2DR: two-drug antiretroviral therapy; pts: patients; HBcAb-positive: patients with antibodies against hepatitis B c antigen; bold numbers if *p*-value < 0.05. ^††^ Data available for 39 pts; ^‡^ Data available for 36 pts.

**Table 4:** Univariate and multivariate analyses for estimated factors with predictive impact on detectable HIV RNA 24 months after the 2DR-3TC-based switch.

**Variables**	**Univariate**		**Multivariate**	
	OR (95% CI)	*p*-value	OR (95%)	*p*-value
Age, years	1.09 (1.05–1.13)	<0.0001	1.08 (1.04–1.13)	**<0.0001**
Calendar year of HIV infection	0.96 (0.91–1.07)	0.10	1.03 (0.97–1.10)	0.25
Nadir CD4+ cell count,/mm^3^	0.99 (0.99–1)	0.083	0.99 (0.99–1)	0.67
2DR-3TC DTG containing	0.93 (0.45–1.92)	0.86	1.16 (0.46–2.9)	**0.74**
HBcAb positivity	3.05 (1.38–6,75)	0.006	2.7 (1.05–6.9)	**0.038**

Bold numbers if *p*-value < 0.05.

## Data Availability

Data available on request from the corresponding author. The data are not publicy available due to privacy restrictions.

## Supplementary Materials

The following are available online at https://www.mdpi.com/2076-2607/9/2/396/s1, Table S1: Comparison of HIV viral load values at the different time points between the HBcAb-positive PLWH and HBcAb-negative PLWH groups switched to 3TC + dolutegravir.
