# Supplementary material for: HBcAb Positivity Is a Risk Factor for an Increased Detectability of HIV RNA after Switching to a Two-Drug Regimen Lamivudine-Based (2DR-3TC-Based) Treatment: Analysis of a Multicenter Italian Cohort
Source: Microorganisms. 2021 Feb 15;9(2):396. doi: 10.3390/microorganisms9020396 (PMC7919011; doi:10.3390/microorganisms9020396)
Supplement: Supplementary file 1 [file microorganisms-09-00396-s001.pdf]

Supplementary table. Comparison of HIV viral load values at the different time points between the PLWH HBcAb-positive and HBcAb-negative groups of patients switched to 3TC+dolutegravir.

	HBcAb-positive (n=34)	HBcAb-negative (n=78)	p-value
Sex ratio M/F, %F	28/6 (17.6%)	56/17 (23.3%)	0.51
Age, years	49 (35-57)	55 (52-59)	**0.0079**
Calendar year of HIV diagnosis, median (range)	2009 (2007-2015)	2004 (1997-2012)	**0.0046**
Nadir CD4+ cell/mmc, median (range)	263 (166-413)	342 (226-466)	0.11
History of therapeutic failure, n (%)	9 (26.3%)	7 (9.6%)	0.12
Pre-2DR switch triple ART composition, n (%):			**0.002**
2NRTI+PI	12 (35.3%)	5 (6.8%)	
2NRTI+NNRTI	9 (26.5%)	23 (31.5%)	
2NRTI+INI	12 (35.3%)	42 (57.5%)	
Other	1 (2.9%)	3 (4.1%)	
CD4+ cell/mmc at 2DR switch, median (range)	781 (565-982)	756 (558-952)	**0.029**
HIV RNA 24 months pre-2DR switch, n (%):			0.89
pts <20 copies/ml	26 (76.5%)	55 (75.3%)	
pts <20 copies/ml target detected	8 (23.5%)	18 (24.6%)	
HIV RNA 12 months pre-2DR switch, n (%):			0.18
pts <20 copies/ml	23 (67.6%)	58 (79.4%)	
pts <20 copies/ml target detected	11 (32.3%)	15 (20.5%)	
HIV RNA at 2DR switch, n (%):			0.63
pts <20 copies/ml	26 (76.5%)	55 (75.3%)	
pts <20 copies/ml target detected	7 (20-6%)	17 (23.3%)	
Not available	1 (3%)	1 (1.4%)	
HIV RNA 6 months post-2DR switch, n (%):			0.12
pts <20 copies/ml	24 (70.6%)	61 (83.6%)	
pts <20 copies/ml target detected	10 (29.4%)	12 (16.4%)	
HIV RNA 12 months post-2DR switch, n (%)*:			**0.02**
pts <20 copies/ml	10 (32.3%)	37 (63.8%)	
pts <20 copies/ml target detected	11 (35.4%)	14 (24.1%)	
pts detectable >20 copies/ml	10 (32.3%)	7 (12.1%)	
HIV RNA 24 months post-2DR switch, n (%)**:			**0.006**
pts <20 copies/ml	5 (20.8%)	26 (54.2%)	
pts <20 copies/ml target detected	9 (37.5%)	16 (33.3%)	
pts detectable >20 copies/ml	10 (41.7%)	6 (12.5%)	

Abbreviations: 3TC: lamivudine; NRTI: nucleoside reverse transcriptase inhibitors; NNRTI: nonnucleoside reverse transcriptase inhibitors; PI: protease inhibitors; DTG: dolutegravir; 2DR: two-drug antiretroviral therapy; pts: patients; HBcAb-positive: patients with antibodies against hepatitis B c antigen * Data available for 81 pts; ** Data available for 72 pts
